# Primary Causes of Death Reported to the FDA Suggest Less Ticagrelor Mortality Benefit than the List Issued to the PLATO Trial Investigators

**DOI:** 10.31083/j.rcm2304132

**Published:** 2022-04-08

**Authors:** Victor Serebruany, Jean-Francois Tanguay, Thomas Marciniak

**Affiliations:** ^1^Department of Neurology, Johns Hopkins University, Baltimore, MD 21218, USA; ^2^Montreal Heart Institute, Interventional Cardiology, Université de Montréal, Montreal, QC H3T 1J4, Canada; ^3^Bethany Beach, DE 19930, USA

**Keywords:** clinical trial, ticagrelor, clopidogrel, cause of death, mortality

## Abstract

**Background:**

The PLATO trial data set reported to the FDA (DRFDA) revealed that some primary deaths causes (PDC) were inaccurately reported favoring ticagrelor. Trial Investigators (DRTI) received different data set with more ticagrelor mortality advantage. We compared these two death lists for the match in PDC.

**Methods and Results:**

The DRFDA contains 938 deaths, while the DRTI contains 905. We matched “vascular”, “non-vascular”, “unknown”, “missed”, and “other” causes of death between DRFDA and DRTI. The DRFDA used 14 vascular, 9 non-vascular, 1 unknown and 1 other PDC codes, while the DRTI used 14 but different vascular, 14 non-vascular but no unknown or other PDC codes. We observed a significant mismatch for the PDC codes between the DRFDA and DRTI data sets. Most DRFDA deaths were vascular (n = 677), fewer non-vascular (n = 159) and unexpectedly many unknown (n = 95) or other (n = 7) PDC. Surprisingly, the shorter DRTI contains more vascular (n = 795), fewer non-vascular (n = 110), but no unknown, other, or missed causes. There were more sudden deaths in DRTI than in DRFDA (161 vs. 138; *p *< 0.03), twice as many post-myocardial infarction deaths (373 vs. 178; *p *< 0.001) but fewer heart failure deaths (73 vs. 109; *p* = 0.02). The reported non-vascular PDC match better except for 2 extra suicides in the clopidogrel arm of the DRTI.

**Conclusions:**

Over 100 “unknown”, “missed”, or “other” PDC events reported by the trial sponsor to the FDA were omitted from the investigator data set contributing to the inflated differences in vascular mortality benefit of ticagrelor reported in numerous PLATO publications. Synchronization of PDC reporting between regulatory agencies and investigators was lacking in PLATO but remains mandatory to ensure quality for future indication-seeking trials.

## 1. Introduction

Ticagrelor is an oral, reversible, direct-acting inhibitor of the adenosine diphosphate receptor $P_{2}$Y12 that was tested against clopidogrel in the PLATO trial. Remarkably for any oral antiplatelet agent, PLATO investigators reported reduced death from vascular causes (4.0% vs. 5.1%, *p* = 0.001), and death from any cause (4.5% vs. 5.9% *p <* 0.001) favoring ticagrelor [1]. While such a strong mortality benefit was surprising, no hard evidence of misreporting has been reported. As of today, ticagrelor holds a superiority recommendation over clopidogrel for acute coronary syndromes in European [2], Canadian [3], and American [4] guidelines based mostly on the official PLATO trial results [1]. We gained access to the detailed data set of 938 PLATO deaths reported by the sponsor to the FDA (DRFDA). We matched the DRFDA to local patient-level data from sites controlled by the sponsor, revealing that actual existence, precise dates and proper primary deaths causes (PDC) in some PLATO patients were inaccurately reported [5]. Several clopidogrel deaths were reported earlier than actual, while their PDC were switched from “non-vascular” or “unknown” to “vascular”. In contrast, few ticagrelor deaths were removed or reported later while some vascular deaths were incorrectly entered in the DRFDA as “non-vascular” or “unknown” [5]. Considering the above-mentioned discrepancies and mismatches, we investigated whether any erroneous records were transferred into the smaller revised PLATO investigators data set (DRTI). Knowing the quality of DRTI is critical because it was used in the original PLATO paper [1] and over 90 peer-reviewed secondary publications with numerous scientific presentations for more than a decade. It was our hypothesis that these two lists would match precisely with the exception of 33 fatalities removed from the broader DRFDA. We repeatedly tried to gain access to the original DRTI for the optimal match [6], but our requests were ignored. However, the PDC data based on DRTI counts were presented by the PLATO Investigators in several good quality papers [7, 8]. In this manuscript, we elucidate whether there were any differences in PDC between these two critical data sets.

## 2. Methods

**Fig. 1.**
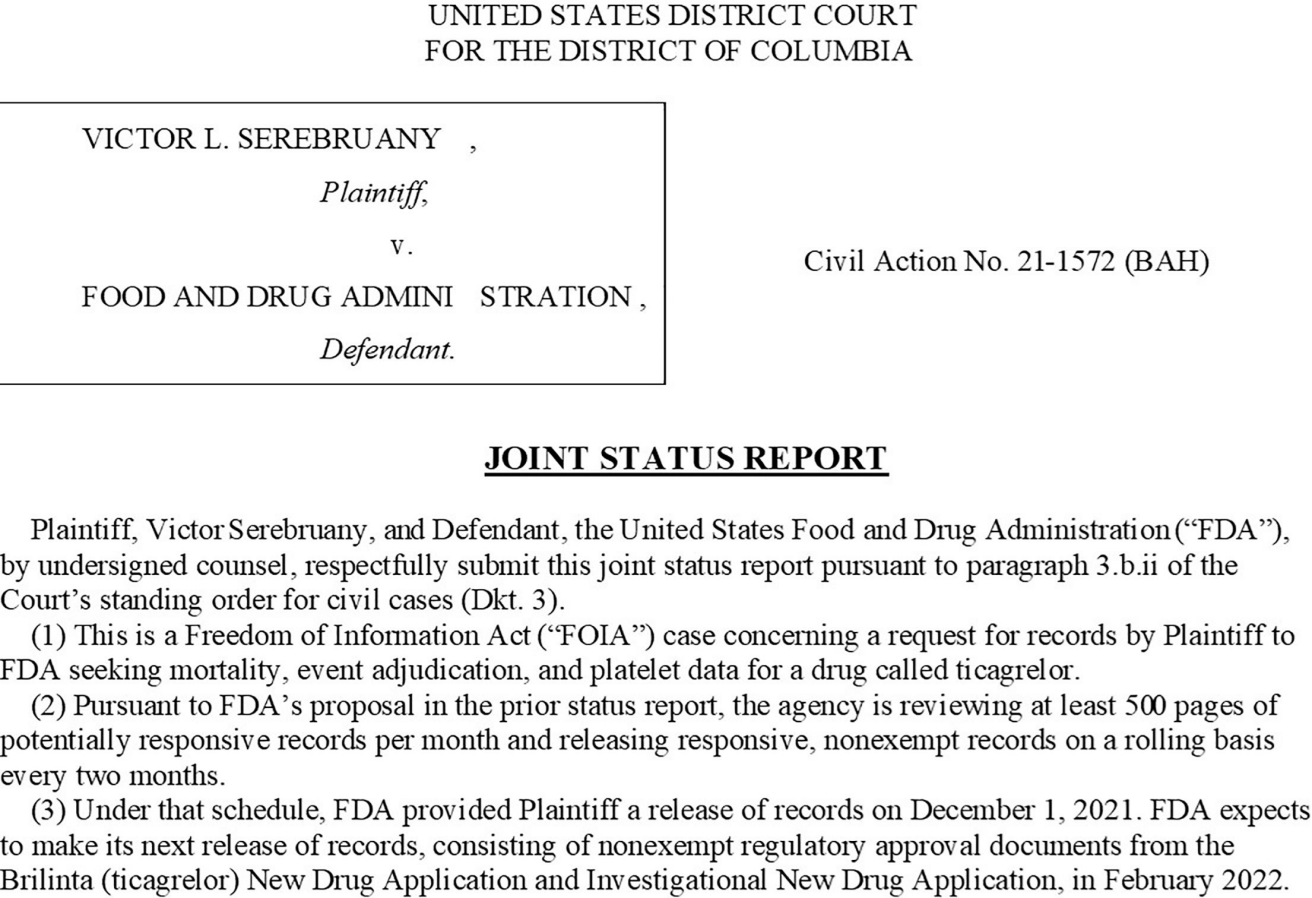
**Front page of the joint status report**.

Based on the Freedom of Information Act, we filed a legal complaint in a US federal court (case 1:21-cv 01572 BAH), reached a joined status report order with FDA and Department of Justice, and received the complete PLATO death data set (DRFDA) submitted to the FDA by the ticagrelor NDA 22-433 sponsor (See Fig. 1 for details).

The DRFDA contains 938 PLATO deaths with trial ID number, country, enrolling site, age, gender, treatment assignment, discontinuation, outcome code, date and precise cause of trial exit. Each record specifies whether the PDC was vascular (code 11), non-vascular (code 12), or unknown (code 97). There were 14 subcodes for vascular (“1” = sudden death; “2” = myocardial infarction; “3” = unstable angina; “4” = other coronary artery disease; “5” = stroke; “6” = arterial embolism; “7” = pulmonary embolism; “8” = ruptured aortic aneurysm; “9” = aortic dissection; “10” = heart failure; “11” = cardiac arrhythmia; “12” = death from bleeding (not trauma); “13” = endocarditis; “14” = valvular disease) 9 subcodes for non-vascular (“1” = respiratory failure; “2” = pneumonia; “3” = cancer; “4” = trauma; “5” = suicide; “6” = liver failure; “7” = renal failure; “8” = sepsis; “9” = multiorgan failure), and a miscellaneous code “99” indicating an “other” vascular or non-vascular PDC. However, the DRTI used different PDC codes. While some of them (sudden, post-AMI (acute myocardial infarction), arrythmia, heart failure, and pulmonary embolism) match, stroke and bleeding codes were expanded, peripheral vascular disease was newly introduced, and unstable angina, other coronary disease, aortic aneurysm or dissection, endocarditis and valvular disease were not assigned. Among non-vascular PDC the codes differences were less prominent. While infections were expanded into 7 categories compared to 2 categories (pneumonia and sepsis) in the DRFDA, other codes match reasonably well. Categorical data are displayed as frequencies and percentages. Statistical analyses were performed using SPSS/11.5 (SPSS, Inc., Chicago, IL, USA).

## 3. Results

**Fig. 2.**
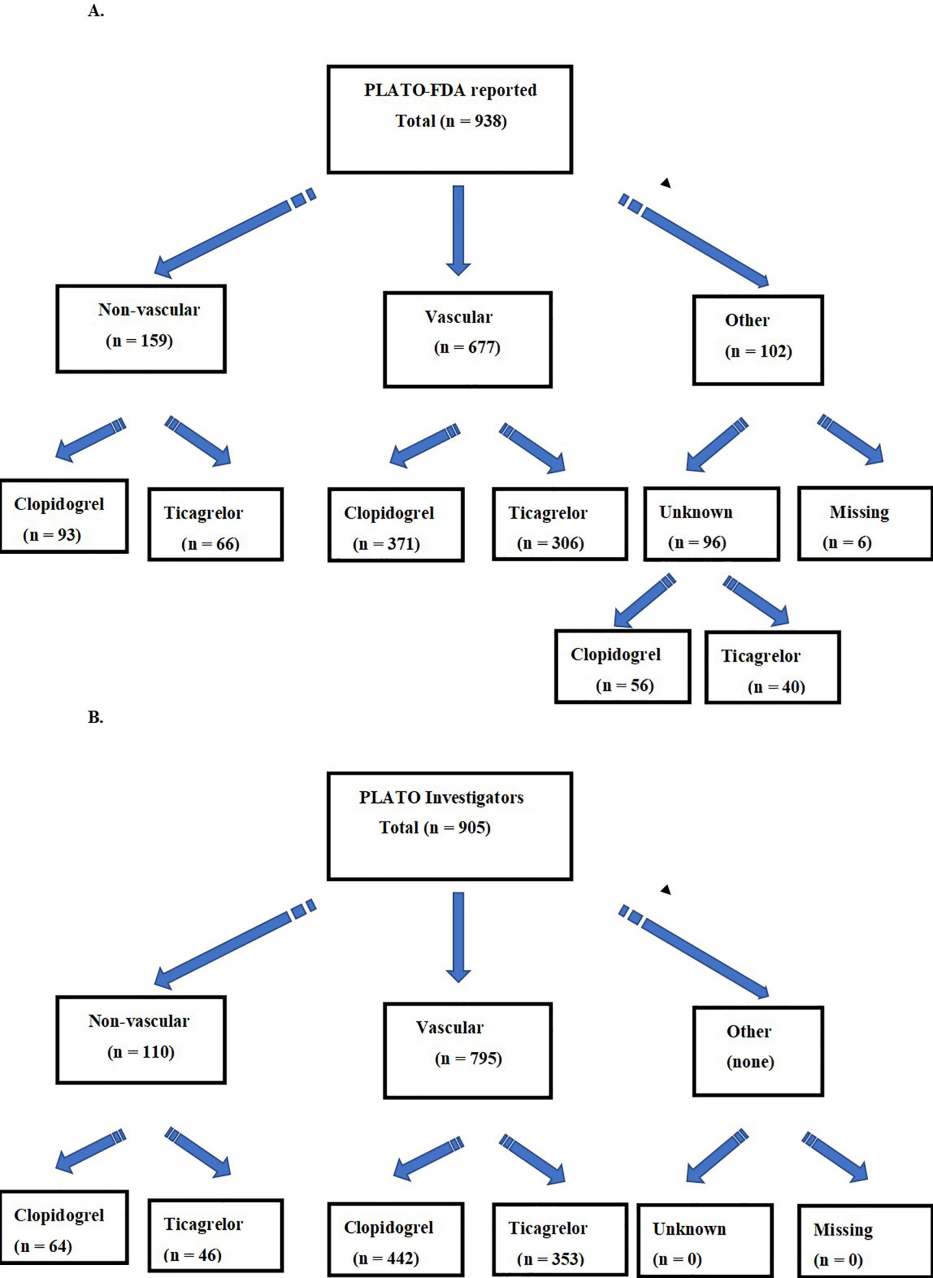
**Flow chart of PLATO primary death causes reported to the FDA (A), and to the Investigators (B)**.

We present in the Fig. 2 the differences in vascular and non-vascular death cause classifications between the DRFDA (A) and the DRTI (B). We show in Table 1 the vascular death causes and in Table 2 the non-vascular death causes for both data sets.

**Table 1.** **Vascular deaths in PLATO**.

Primary death cause	PLATO code	DRFDA-C	DRFDA-T	DRTI-C	DRTI-T
		(n = 371)	(n = 306)	(n = 442)	(n = 353)
Sudden	11-1	78	60	98	63
Myocardial infarction	11-2	90	88	194	179
Unstable angina	11-3	8	7	NR	NR
Other CAD	11-4	4	4	NR	NR
Stroke	11-5	18	21	18	19
Arterial embolism	11-6	2	-	NR	NR
Pulmonary embolism	11-7	8	2	5	-
Ruptured aortic aneurism	11-8	-	1	-	NR
Aortic dissection	11-9	2	1	NR	NR
Heart failure	11-10	62	47	42	31
Cardiac arrhythmia	11-11	26	20	5	2
Bleeding (not trauma)	11-12	17	12	17	10
Endocarditis	11-13	-	-	-	-
Valvular disease	11-14	1	-	NR	-
Other	99	55	43	8	6

CAD, coronary artery disease; C, clopidogrel; T, ticagrelor; NR, not reported.

**Table 2.** **Non-vascular deaths in PLATO**.

Death cause	PLATO code	DRFDA-C	DRFDA-T	DRTI-C	DRTI-T
		(n = 93)	(n = 66)	(n = 64)	(n = 46)
Respiratory failure	12-1	12	12	NR	NR
Pneumonia	12-2	8	10	NR	NR
Cancer	12-3	17	14	17	15
Trauma	12-4	1	3	1	3
Suicide	12-5	1	1	3	1
Liver failure	12-6	1	-	2	1
Renal failure	12-7	5	2	3	0
Sepsis	12-8	23	7	23	7
Multiorgan failure	12-9	14	9	5	3
Other	99	11	8	2	1

C, clopidogrel; T, ticagrelor; NR, not reported.

Both data sets identify a single PDC, which we would expect to be identical [5, 6, 7]. The PDC may be “unknown” or “other” but there should be just one PDC. Regarding vascular deaths there are significant differences between the DRFDA and the DRTI (677 vs. 795; *p *< 0.001). There are more sudden deaths in the shorter DRTI list than in the DRFDA (161 vs. 138; *p *< 0.03) and post-AMI deaths (373 vs. 178; *p *< 0.001) but less heart failure deaths (73 vs. 109; *p* = 0.02). Stroke numbers match well (39 vs. 37; *p* = NS) with only 2 ticagrelor cases removed. Among non-vascular causes of deaths, cancer deaths match well (32 vs. 31; *p* = NS), and sepsis cases were identical (30 vs. 30; *p *= NS), but 2 extra suicide cases after clopidogrel in DRTI could deserve special attention. Also notable is the substantial reduction of “other” causes for both vascular and non-vascular deaths, and the total elimination of “unknown” PDC from the DRTI dataset.

## 4. Discussion

The main finding of this report is that PLATO sponsor issued different death data sets to the FDA and to the trial investigators. Matching two supposedly very similar data sets reveals significant discrepancies in reported PDC. Aside from acceptable, expected and conventional reduction or “cleaning” from the broader DRFDA list to the final DRTI dataset, there could be other reasons for such modifications in PDC that could lead to an artificially enhanced ticagrelor benefit. In fact, over one hundred ‘unknown or missing’ PDC cases were readjudicated into the vascular death category favoring ticagrelor in DRTI. It is unclear which fatalities were readjudicated, or how and by whom the PDC were finally defined in DRTI. Why the death data sets do not match and why this discrepancy has remained unreported by regulatory authorities for over a decade is also unclear.

Obviously, as in any phase 3 trial, deceased patients will experience multiple diseases, comorbidities, and/or severe associated conditions contributing to death. All of them should be properly reported in the electronic Clinical Research Form, but PLATO clearly specified that only a single coded PDC matters and should be counted for the efficacy endpoint, that is, one patient, one cause. The trial primary outcome depended upon this unary assignment. The expansion of sudden deaths as PDC for the clopidogrel arm in the investigators data set deserves special attention. It was the exact task of the PLATO Central Adjudication Committee (CAC) to identify the single proper cause of death, fairly assessing the existing decision of the local site report [9], and reporting both identically in the DRTI and the DRFDA. The pivotal role of the CAC or other party responsible for the data set handling leading to extra myocardial infarctions delegated exclusively to the clopidogrel arm in PLATO [10, 11] is also of concern. Such discrepancies in data sets and the role of the clinical event adjudicating are matters of ongoing investigation by our Task Force to maintain the validity of future clinical trials.

With regard to the mismatch in total PLATO deaths, it is our current understanding that the data set we have in possession was later restricted or reduced, while some deaths reported to the FDA occurred after the trial ended. But an absolute majority (over 900) of deaths should match precisely between the two data sets. We identified several controversies when the patient never received a single drug dose, heavy enrollment mistakes, and inclusion/exclusion errors, but the most common restriction was the length of follow-up. The PLATO death count was strictly limited to no more than 365 days follow-up duration [9]. In fact, any death within the trial period should be counted although PLATO also excluded deaths after “withdrawal of consent” or “volunteer discontinuations” mostly for ticagrelor patients [9]. Importantly, there were some PLATO patients who were still on drug, died 12–16 months after enrollment, while the trial was still active at the second half of 2008, but these patients were excluded as being beyond the trial time frame. Finally, we remain skeptical that the DRFDA (n = 938) reductions resulted in the removal of more ticagrelor (n = 21) than clopidogrel (n = 12) entries for the final count of 905 deaths in the DRTI.

Any description of how documents flowed back and forth between sites, sponsor, investigators, adjudicators, academic research organizations, data safety monitoring board members, pharmacovigilance monitors is lacking, and these interactions remain in the “grey” zone. The FDA reviewers occasionally got some documents, e.g., what the DSMB saw, and some adjudication files. However, what the FDA typically gets are analytic data sets, likely ones also used by the investigators, that reflect the adjudications—and likely post processing by the sponsor. Importantly, the deaths should be the same as the counts in the publications. The FDA also usually gets files roughly corresponding to the clinical research forms. They should contain almost all deaths, including the late ones. In PLATO, the CAC got files periodically with the events to be adjudicated. The CAC then returned adjudications to the sponsor. What happened after is still unclear, but there is the possibility for adjustments.

Finally, suicides deserve special attention. Two extra cases [7] in the shorter DRTI are difficult to comprehend. Not only is suicide (PLATO code 12-5) a valid PDC, which cannot be substituted, but both “missing” patients are from the clopidogrel arm. We directly approached PLATO Investigators with detailed specific inquiries regarding these 2 suicides unreported to the FDA, but we received no response or any explanation on these 2 extra deaths in the DRTI [6]. Not solving such a simple issue not only raises questions regarding inaccurate vascular deaths but also challenges the claimed all-cause mortality benefit of ticagrelor, which has not been observed in later clinical trials.

As of today, the evidence suggests that the DRFDA and DRTI datasets for PLATO were different, and significantly mismatched. Importantly, 905 deaths reported in almost 100 of papers in leading journals by PLATO Investigators based on the DRTI should be identical to those listed in the DRFDA. Moreover, the deaths classification codes applied were different as well, so it remains unclear why such efforts were not synchronized. However, it may appear that death causes, especially vascular ones, were significantly reclassified further, before the DRFDA submission. Alternatively, it could be possible that the two separate death data sets existed simultaneously since the chronology of events does not support the fact that the shorter DRTI list was derived from the broader DRFDA dataset. In fact, the investigators published the main PLATO paper [1] (September 10, 2009) earlier than the submission to the FDA of the ticagrelor New Drug Application (November 16, 2009) [9]. One inaccurate death data set could have been provided to the FDA for gaining regulatory approval, while a different dataset with even greater ticagrelor benefit could have been presented to trial investigators. There are no limitations to our report offering any reasonable alternative explanations to such observed mismatch in datasets. The DRTI 905 deaths are reported identically among all related PLATO publications [1, 7, 8] and the expanded PDC-different list of 938 deaths was issued directly by the FDA to our Task Force overseen by the US Department of Justice. Over 100 of adjudicated as “unknown” PDC lacking in DRTI but reported to the FDA could have used as a “pool” for the vascular mortality “benefits” of ticagrelor magnification in overoptimistic publications and presentations by PLATO Investigators.

## 5. Conclusions

Over 100 “unknown”, “missed”, or “other” PDC events reported by the trial sponsor to the FDA were omitted from the investigator data set contributing to the inflated differences in vascular mortality benefit of ticagrelor later reported in dozens of PLATO publications. Synchronization of PDC reporting between regulatory agencies and investigators was lacking in PLATO but remains mandatory to ensure quality for future indication-seeking trials.
